# e-Pharmacophore modeling and *in silico* study of CD147 receptor against SARS-CoV-2 drugs

**DOI:** 10.5808/gi.23005

**Published:** 2023-06-30

**Authors:** Nisha Kumari Pandit, Simranjeet Singh Mann, Anee Mohanty, Sumer Singh Meena

**Affiliations:** Department of Biotechnology, Dr. B. R. Ambedkar National Institute of Technology Jalandhar, Punjab 144027, India

**Keywords:** CD147, CDOCKER, COVID-19, e-pharmacophore, ritonavir, SARS-CoV-2

## Abstract

Coronavirus has left severe health impacts on the human population, globally. Still a significant number of cases are reported daily as no specific medications are available for its effective treatment. The presence of the CD147 receptor (human basigin) on the host cell facilitates the severe acute respiratory disease coronavirus 2 (SARS-CoV-2) infection. Therefore, the drugs that efficiently alter the formation of CD147 and spike protein complex could be the right drug candidate to inhibit the replication of SARS-CoV-2. Hence, an e-Pharmacophore model was developed based on the receptor-ligand cavity of CD147 protein which was further mapped against pre-existing drugs of coronavirus disease treatment. A total of seven drugs were found to be suited as pharmacophores out of 11 drugs screened which was further docked with CD147 protein using CDOCKER of Biovia discovery studio. The active site sphere of the prepared protein was 101.44, 87.84, and 97.17 along with the radius being 15.33 and the root-mean-square deviation value obtained was 0.73 Å. The protein minimization energy was calculated to be –30,328.81547 kcal/mol. The docking results showed ritonavir as the best fit as it demonstrated a higher CDOCKER energy (–57.30) with correspond to CDOCKER interaction energy (–53.38). However, authors further suggest *in vitro* studies to understand the potential activity of the ritonavir.

## Introduction

Coronaviruses belong to Coronaviridae family which causes severe respiratory diseases in human beings. These viruses were initially isolated from a human in the year 1965 and are positive-sense, single-stranded RNA viruses. Until now, there are three major outbreaks caused due to coronavirus, namely, severe acute respiratory syndrome coronavirus (SARS-CoV) in the year 2002, Middle East respiratory syndrome coronavirus (MERS-CoV) in the year 2012, and recently, SARS-CoV-2 in the year 2019 [1]. The recent pandemic of coronavirus disease 2019 (COVID-19) that emerged in December 2019 has affected the whole nation and has put restrictions on the movement of people. It has been cautioned to every individual globally to remain at home and not to assemble, wash hands regularly, use face mask and hand sanitizer and keep physical distance [2]. SARS-CoV-2 which triggers the coronavirus disease is considered to have originated in the laboratories of Wuhan, China. It is the seventh human coronavirus that can cause severe health issues, majorly infecting the lungs that can eventually cause death. The frequently reported symptoms of COVID include shivering with high body temperature, cough, and difficulty breathing [3].

Currently, there are no relevant medications available for effective treatment of coronavirus. Drug repurposing has shown positive effects but they are incapable of preventing the disease. To treat coronavirus, it is very important to identify the therapeutic target or a drug that efficiently arrests the replication of SARS-CoV-2 [4]. One of the methods can be finding an antiviral drug or a molecule that can interfere with the spike protein or CD147 expression [5].

There are mainly two receptors that could be pathways for SARS-CoV-2 to get inside the host cell, namely angiotensin-converting enzyme 2 (ACE2) and CD147. The spike protein present externally on coronavirus binds with either ACE2 or CD147 receptor, thus intervening capture and spreading of the virus to another cell [5]. Recently, Wang et al. [6] illustrated that the expression level of ACE2 is low and there may be other routes for viral entry which later they found to be CD147. They came across the activity of the spike protein of SARS-CoV-2 and CD147 receptor-mediated by meplazumab which is an anti-CD147 humanized antibody. The use of this antibody blocks the host cell receptor CD147 which in turn inhibits the amplification of SARS-CoV-2. This particular work revealed an important target that can be utilized in developing a therapeutic drug against COVID-19 [7]. Furthermore, the study conducted by Geng and a co-worker revealed CD147 as a universal receptor for SARS-CoV-2 as well as its other variants [8].

CD147 or basigin or extracellular matrix metalloproteinase inducer is an integral membrane glycoprotein that comprises mainly two immunoglobulin units present in the extracellular region. The two domains of immunoglobulin are a single transmembrane domain and a cytoplasmic domain [9]. Apart from facilitating SARS-CoV infection, it also facilitates tumor growth, *Plasmodium* capture, and infection of viruses and bacteria [7]. CD147 is thought to regulate cytokine production and leucocyte chemotaxis when it binds to cyclophilin A (CyPA) [10]. Cyclophilins belong to the PPlase family of intracellular proteins which is highly conserved. It is the most commonly expressed protein of this family consisting of seven types of which CyPA is the most abundant and dominant protein [4].

This work aims to design and identify inhibitors of human basigin to stop viral entry into the cell by developing an e-Pharmaco­phore model of receptor-ligand interaction between CD147 and various U.S. Food and Drug Administration (FDA)–approved drugs for COVID-19. Pharmacophore modeling is a part of computer-aided drug design (CADD) that plays a key role when there is no structure of receptor presents [11]. Pharmacophores may be described as the arrangement of a molecule that stores the essential characteristics necessary for the biological or pharmacological interaction of drugs. It is used for screening the molecules virtually that triggers a biological response. A pharmacophore model usually has two procedures, ligand-related and structure-related [12]. In ligand-related pharmacophores modeling, a set of active molecules are superposed in order to get the chemical activities that are necessary for biological activities [13]. However, in structure-related method a pharmacophore model is developed using the structural features of the protein. In this model, the interaction between ligands and macromolecular targets is taken into account [14].

The technique of molecular docking has been widely applied for many decades in the field of drug discovery and many other aspects of bioinformatics. The method takes into account the orientation of a molecule and how the molecule changes its conformation after binding to a particular molecular target [15]. It is a structure-based drug design where binding occurs between a receptor and a ligand. There are various software developed to date, among which are some well-known, AutoDock, AutoDock Vina, ZDOCK, RDOCK, Glide, and Gold [16]. The first step in molecular docking involves generating a three-dimensional structure of a ligand and receptor from various databases available like Protein Data Bank (PDB) or can be drawn using tools e.g., ChemSketch, and preparing it. The next step involves defining and calculating of grid where the ligand can be docked. And finally, the docking process can be carried out and the results can be visualized using a visualization software [17].

## Methods

**Fig. 1.**
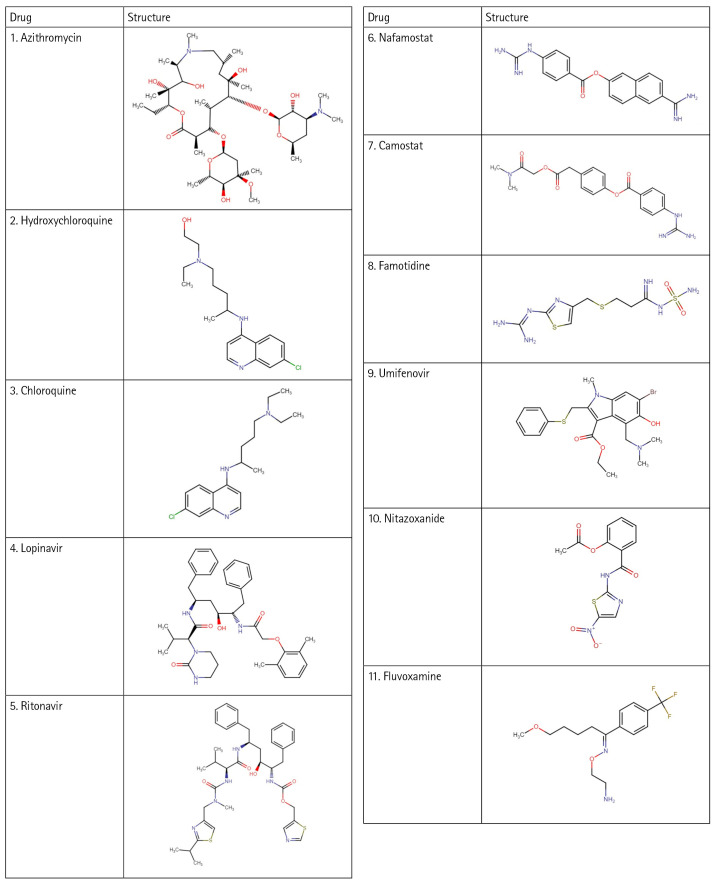
Structure of drugs.

In this work, with the help of the Biovia Discovery Studio platform, an e-pharmacophore model was developed using the protein Human Basigin (PDB ID 7DCE) in a structure-based manner. The developed model was used for screening the drug targets. A total of eleven drugs (Fig. 1), drawn online using Chem-Space were used for screening. These drugs were selected based on the literature studies [18,19] that have shown activity in COVID-19 therapy. All the drugs act differently and have several mechanisms against coronavirus. Various *in vitro* studies showed the drugs to be helpful in blocking the viral production of this deadly virus. Therefore, these drugs have been utilized for repurposing studies and to understand their ability to block the CD147 receptor to treat COVID-19. Further, the reasons for selecting these eleven drugs are described below.

### Drugs

#### Azithromycin

Azithromycin is an FDA-approved drug which is mainly used to treat respiratory diseases like pneumonia. A person suffering from COVID-19 shows the symptoms of pneumonia and acute respiratory problems where azithromycin presents a promising drug candidate [20]. This drug has antibacterial properties along with the properties of antiviral and immunomodulatory that could be an interest in the treatment of COVID-19. It is a bacteriostatic antibiotic showing *in vitro* activity against SARS-CoV-2 and acts on the viral cycle at different points [21].

#### Hydroxychloroquine

This medication has been used for a long period of time for the treatment of malaria. Apart from its antimalarial effects, it also shows antiviral and anti-inflammatory effects. Since hydroxychloroquine shows its effectiveness across a broad range of diseases, it is also thought to be useful in viral infection [22]. It also has certain metabolic benefits which include lipid profiles and lower blood glucose levels. The pharmacokinetics of this drug vary among different individuals and its oral bioavailability is about seventy-five percent [23].

#### Chloroquine

Recently, the benefits of chloroquine have been observed in treating the symptoms of COVID-19. Alike, hydroxychloroquine this drug also has antimalarial and antiviral effects [24]. Chloroquine phosphate and sulfate, both have been made commercially available in the market as antimalarial drugs of which chloroquine phosphate proved to be beneficial in treating COVID-19-associated pneumonia [25].

#### Lopinavir

Lopinavir belongs to the class of protease inhibitors which is an antiretroviral drug. It is usually used along with ritonavir for the treatment and therapy of human immunodeficiency virus (HIV) infections. This medication shows activity in the treatment of COVID-19 as it inhibits the protease activity of coronavirus. It also stops the replication cycle of MERS-CoV as it blocks the post-entrance steps [26].

#### Ritonavir

Ritonavir, along with lopinavir is used to treat HIV infections. This medication is marketed using the name Norvir and it behaves as a protease inhibitor. Ritonavir, when combined with lopinavir acts as an inhibitor of drug metabolism whereas lopinavir acts as a principal antiviral compound. Another compound named Atazanavir, when used in combination with ritonavir showed successful activity in hindering the SARS-CoV-2 replication cycle [27].

#### Nafamostat

This medication belongs to the group of serine protease inhibitors, majorly used for therapy of dispersed intraventricular coagulation and pancreatitis. Various publications and *in vitro* studies suggests that this medication is beneficial for curing pneumonia in individual suffering from coronavirus disease [28].

#### Camostat

Camostat mesylate is a drug available for oral consumption. It belongs to the group of serine protease inhibitors which potentially degrades transmembrane serine protease 2. This drug is hypothesized to be potentially used against the coronavirus. It mainly inhibits the fusion of virus and cell membrane which in turn inhibits the replication of the virus [29].

#### Famotidine

Famotidine is over a counter drug which is widely used to treat gastric acid. This drug is safe to use and does not react with any other drug. It is a histamine 2 receptor antagonist and can be presented as a candidate for the therapy of coronavirus. The study conducted by Janowitz and co-author on patients suffering from COVID-19 suggests that high doses of famotidine can prove to be beneficial for the treatment of coronavirus [30].

#### Umifenovir

Umifenovir (Arabidol) is an antiviral medication mainly used for treating the infection caused by influenza viruses as it targets the membrane fusion process of influenza virus. Arabidol therapy or Arabidol in combination with darunavir is thought to be a possible approaches for treating the deadly coronavirus [31]. This drug prevents the integration of the virus lipid cell with the plasma membrane which restricts the virus from entering the host cell. This drug is thought to inhibit coronavirus infection as it interferes with the release of SARS-CoV-2 from intracellular vesicles [32].

#### Nitazoxanide

Nitazoxanide is a drug that is used to treat infection caused by protozoan, for example, diarrhea which is caused by the protozoa *Cryptosporidium* and *Giardia*. This drug has also been efficient against broad range of viruses *in vitro* including rotavirus, norovirus, hepatitis B virus, and dengue virus. It has shown an acceptable *in vitro* activity against the coronavirus that makes it a probable candidate for treating SARS-CoV-2 [33].

#### Fluvoxamine

Fluvoxamine is a selective serotonin reuptake inhibitor used for the treatment of the obsessive-compulsive disorder. It decreases cytokine production by activating the sigma-1 receptor that resides inside the endoplasmic reticulum of the cell. This drug proves to be a promising approach for the advanced treatment of COVID [34].

### Protein selection and preparation

**Fig. 2.**
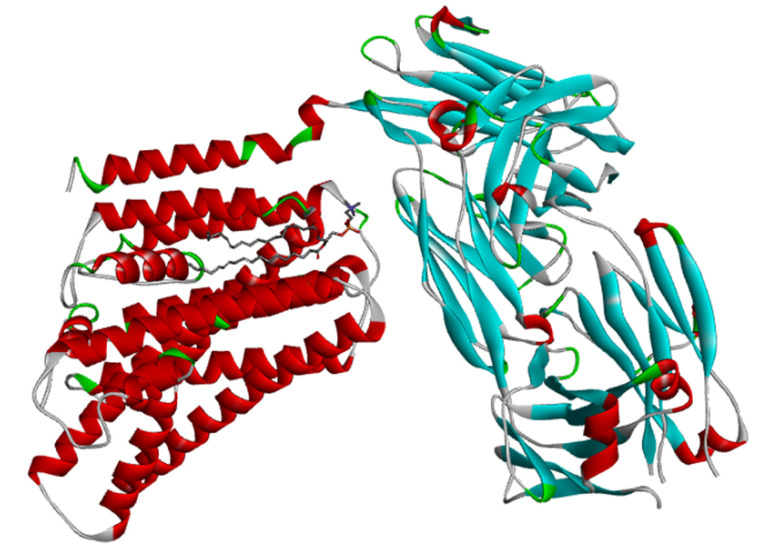
Cryogenic electron microscopy structure of human XKR8-Basigin complex bound to Fab fragment; PDB ID-7DCE.

Fig. 2 shows the cryogenic electron microscopy (cryo-EM) structure of the human XKR8-Basigin complex bound to Fab fragment (PDB ID 7DCE) which was retrieved from PDB at a resolution of 3.80 Å. This protein is bound to a Fab fragment which will be utilized further to produce an e-pharmacophore model. The selected protein was firstly used for pharmacophore generation and later it was prepared for docking. Thereafter, it was subjected to energy minimization by applying CHARMm force field, and also the root-mean-square deviation (RMSD) value was calculated.

### Pharmacophore generation

The first step in the generation of the pharmacophore model is removing the ligand present in the protein. After removing the ligand, an e-Pharmacophore model was developed in a structure-based manner utilizing the Biovia discovery studio platform. Later, this model was used to screen the above-mentioned eleven drug targets.

### Ligand preparation

A total of seven drug targets (chosen after screening with the e-pharmacophore model) were presented to dock against the protein molecule as these are used for treating the symptoms of COVID-19. All the ligands were prepared to dock against the prepared protein.

### Protein ligand docking

To begin with molecular docking, the first and foremost step was determining the site sphere of the protein to know our ligand binding site. Thereafter, the prepared protein and different ligands were docked using CDOCKER tool of Biovia Discovery Studio. CDOCKER (CHARMm-based DOCKER) tool utilizes CHARMm’s position for producing highly accurate docking results as it provides full flexibility for the ligand which includes bonds, dihedrals, angles, thereby generating the various conformations of the docked receptor and ligand. Apart from advantageous ligand flexibility, CDOCKER tool also offers CHARMm family force fields, flexibile CHARMm engine, and efficient time for computation analysis [35]. The CDOCKER energy is taken into account to find the best conformations amongst various refined poses that were generated.

## Results

### e-Pharmacophore model

**Fig. 3.**
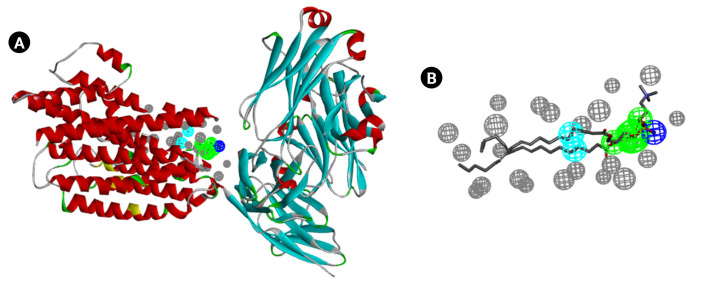
(A) Generated e-Pharmacophore model showing the pockets where ligands can be fitted. (B) Generated e-Pharmacophore model without protein.

Fig. 3A and 3B show the e-pharmacophore model that was developed using the Biovia Discovery Studio platform. Nineteen non-bonded interactions were found between the ligand and the receptor and a total of 10 pharmacophores were generated.

### Protein preparation and minimization

The active site sphere of the prepared protein was 101.44, 87.84, and 97.17 along with the radius being 15.33. After superimposing the original protein with prepared protein an acceptable RMSD value of 0.73 Å was obtained which confirmed that the prepared protein structure is similar to that of original protein. The protein minimization energy obtained was –30,328.81547 kcal/mol. The following values of different energies were generated, bond energy 651.327, angle energy 3,287.27, dihedral energy- 2,970.2, Vanderwaal energy –6,016.41, electrostatic energy –54,180.5, and the value of CHARMm was obtained as –52,832.1.

### CDOCKER results

All eleven drug targets were screened and only seven of them fitted the pharmacophore.

#### Hydroxychloroquine

**Fig. 4.**
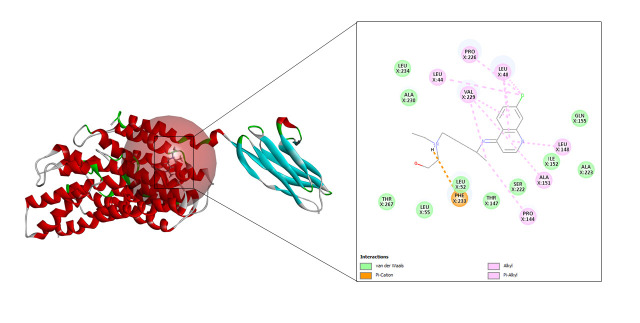
CD147-hydroxychloroquine interaction.

After docking the prepared protein with hydroxychloroquine, 40 refined poses were generated in total of which three were selected based on docking score shown in Table 1. Fig. 4 depicts the best interaction that had the highest CDOCKER energy and CDOCKER interaction energy of –20.7452 and –35.2816 respectively.

#### Lopinavir

**Fig. 5.**
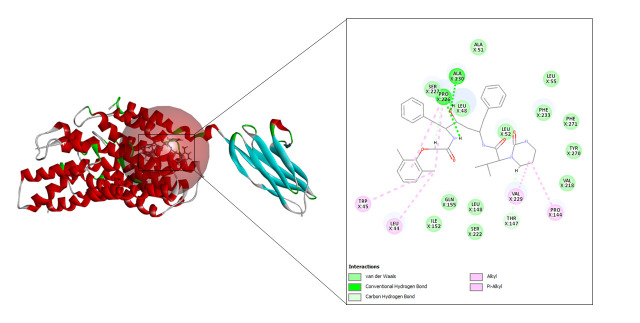
CD147-lopinavir interaction.

A total of 10 refined poses were generated after docking with lopinavir. The top three docking values are shown in Table 2 and the best fit is depicted in Fig. 5 as it displayed the highest CDOCKER energy and CDOCKER interaction energy of –45.0255 and –57.952 respectively.

#### Ritonavir

**Fig. 6.**
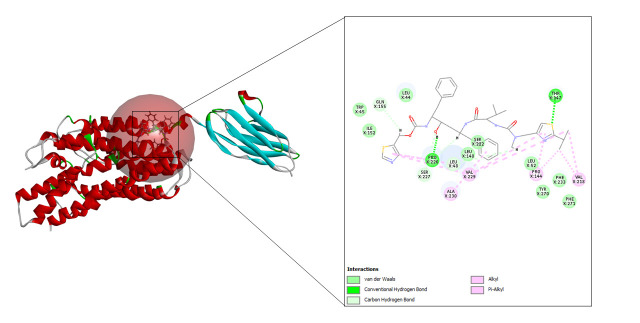
CD147-ritonavir interaction.

In case of ritonavir, 20 refined poses were generated and the top three ranked are depicted in Table 3. The highest CDOCKER energy and CDOCKER interaction energy generated was –57.3072 and –53.3876 respectively shown in Fig. 6.

#### Camostat

**Fig. 7.**
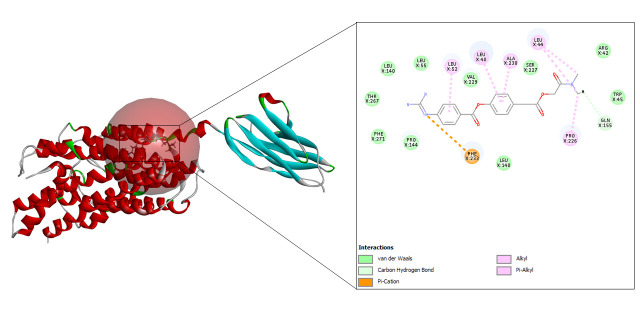
CD147-camostat interaction.

Docking with camostat resulted in the generation of 20 refined poses and the top three are depicted in Table 4. The best fit, shown in Fig. 7 had the highest CDOCKER energy and CDOCKER interaction energy of –20.3898 and –35.6218 respectively.

#### Famotidine

**Fig. 8.**
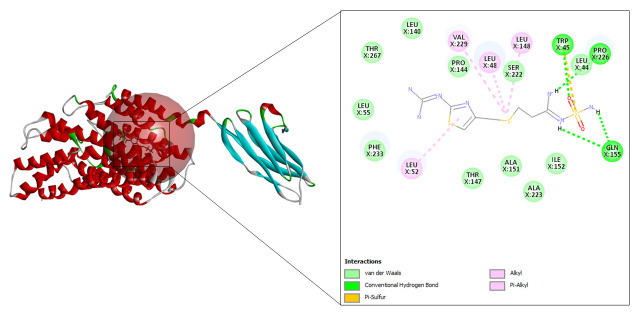
CD147-famotidine interaction.

Famotidine generated the highest refined poses amongst all which is 110 poses. The top three docking values is given in Table 5 and Fig. 8 displays the best fit of the highest CDOCKER energy and CDOCKER interaction energy which is –26.8616 and –28.8008 respectively.

#### Umifenovir

**Fig. 9.**
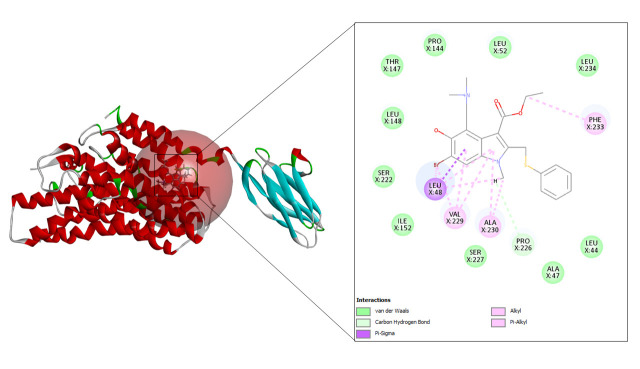
CD147-umifenovir interaction.

Upon docking the prepared protein with umifenovir, a total of 20 refined poses were generated amongst which the top three docking values are shown in Table 6. Fig. 9 shows the best-fit pose having the highest CDOCKER energy and CDOCKER interaction energy of –25.1386 and –35.2122, respectively.

#### Fluvoxamine

**Fig. 10.**
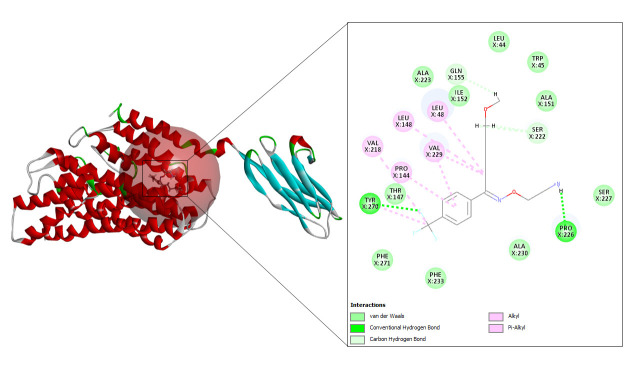
CD147-fluvoxamine interaction.

Fluvoxamine also resulted in 110 refined poses, as in case of famotidine. The top three docking values are shown in Table 7 and the best fit is depicted in Fig. 10 as it displayed the highest CDOCKER energy and CDOCKER interaction energy of –29.8226 and –39.2161, respectively.

## Discussion

The cryo-EM structure of human XKR8-Basigin complex bound to Fab fragment (PDB ID 7DCE) was acquired from Protein Data Bank at a resolution of 3.80 Å. An e-pharmacophore model was developed using this protein (7DCE) to generate pockets where drug targets can fit accordingly. Eleven drugs, namely, azithromycin, hydroxychloroquine, chloroquine, lopinavir, ritonavir, nafamostat, camostat, famotidine, umifenovir, nitazoxanide, and fluvoxamine were considered for this work as these drugs are currently being utilized for the treating the symptoms of coronavirus. These drugs after preparation were screened in the pharmacophore model amongst which seven of them, viz, hydroxychloroquine, lopinavir, ritonavir, camostat, famotidine, umifenovir, and fluvoxamine fitted to the pharmacophore pockets. The rest of the drugs i.e., azithromycin, chloroquine, nafamostat, and nitazoxanide could not screen as these drugs do not interfere with the CD147 receptor. Later, the protein was prepared and energy minimization was performed to determine a proper molecular arrangement. After defining the site sphere molecular docking was performed between the receptor (human basigin) and the screened drug targets using CDOCKER. According to the CDOCKER energy generated, ritonavir emerged as the best drug demonstrating a higher CDOCKER energy of –57.30 and its corresponding CDOCKER interaction energy of –53.38, respectively. In summary, our results contribute to the findings of relevant drugs for COVID-19 and can be of great importance for other scientific works in the future.

## Figures and Tables

**Table 1.** -CDOCKER energy and -CDOCKER interaction energy of hydroxychloroquine-CD147 complex

	-CDOCKER_ENERGY	-CDOCKER_INTERACTION_ENERGY
?	20.7451	35.2816
?	19.9686	35.973
?	16.7187	30.459

**Table 2.** -CDOCKER energy and -CDOCKER interaction energy of lopinavir-CD147 complex

	-CDOCKER_ENERGY	-CDOCKER_INTERACTION_ENERGY
?	45.0255	57.952
?	42.0827	44.1381
?	42.0644	44.4358

**Table 3.** -CDOCKER energy and -CDOCKER interaction energy of ritonavir-CD147 complex

	-CDOCKER_ENERGY	-CDOCKER_INTERACTION_ENERGY
?	57.3072	53.3876
?	54.152	49.1827
?	53.9684	47.3915

**Table 4.** -CDOCKER energy and -CDOCKER interaction energy of camostat-CD147 complex

	-CDOCKER_ENERGY	-CDOCKER_INTERACTION_ENERGY
?	20.3898	35.6218
?	20.2663	35.175
?	20.0669	36.949

**Table 5.** -CDOCKER energy and -CDOCKER interaction energy of famotidine-CD147 complex

	-CDOCKER_ENERGY	-CDOCKER_INTERACTION_ENERGY
?	26.8616	28.8008
?	26.5134	29.4275
?	26.3377	28.1334

**Table 6.** -CDOCKER energy and -CDOCKER interaction energy of umifenovir-CD147 complex

	-CDOCKER_ENERGY	-CDOCKER_INTERACTION_ENERGY
?	25.1386	33.2122
?	25.0374	33.6277
?	24.906	33.8427

**Table 7.** -CDOCKER energy and -CDOCKER interaction energy of fluvoxamine-CD147 complex

	-CDOCKER_ENERGY	-CDOCKER_INTERACTION_ENERGY
?	29.8226	39.2161
?	29.3781	34.895
?	28.8813	35.1303
